# Effects of Second-Generation Long-Acting Injectable Antipsychotics on Brain Structure in Schizophrenia: A Systematic Review of Neuroimaging Studies

**DOI:** 10.3390/brainsci16101020

**Published:** 2026-09-25

**Authors:** Vassilis Martiadis, Pasquale Scognamiglio, Fabiola Raffone, Miriam Olivola, Valeria Iniziato, Tommaso Barlattani, Francesca Pacitti, Domenico De Berardis

**Affiliations:** 1 Department of Mental Health, ASL Napoli 1 Centro, 80145 Naples, Italy; 2 National Institute for Social Security (INPS), Via Medina 61, 80133 Naples, Italy; 3 Department of Psychiatry, University of Campania “Luigi Vanvitelli”, Largo Madonna delle Grazie 1, 80138 Naples, Italy; 4 Department of Mental Health and Addiction Services, ASST Fatebenefratelli-Sacco, 20157 Milan, Italy; miriam.olivola@asst-fbf-sacco.it; 5 Department of Biotechnological and Applied Clinical Sciences (DISCAB), University of L’Aquila, Via Vetoio, Coppito, 67100 L’Aquila, Italyfrancesca.pacitti@univaq.it (F.P.); 6 Department of Mental Health, ASL 4 Teramo, 64100 Teramo, Italy; domenico.deberardis@aslteramo.it

**Keywords:** LAI, second generation long-acting, antipsychotics, MRI, DTI, functional MRI, neuroimaging, neuroprotection, gray matter, white matter, myelination

## Abstract

**Highlights:**

**What are the main findings?**

- Across nine neuroimaging studies, second-generation long-acting injectable antipsychotics were associated with better preservation of intracortical myelin and of cortical thickness than oral formulations, most consistently in frontal regions, and two cross-sectional fMRI studies described activation patterns closer to those of healthy controls than in patients treated with first-generation depots.
- Two features of this literature emerged that had not been quantified before: the nine studies correspond to only five independent samples, and myelin-sensitive and diffusion-based measures moved in opposite directions within the same randomized cohort.

**What are the implications of the main findings?**

- The structural signal is consistent enough to be worth testing directly, and it gives clinicians a concrete biological argument to discuss with patients when an injectable formulation is proposed early in the illness, provided it is presented as a plausible effect still under investigation.
- Trials should prespecify imaging endpoints alongside clinical and cognitive outcomes, report myelin-sensitive and diffusion-based measures together, recruit cohorts independent of the two programmes that currently dominate the field, and extend the evidence beyond risperidone and paliperidone.

**Abstract:**

**Background/Objectives:** Second-generation long-acting injectable antipsychotics (SGA-LAIs) are increasingly used to improve adherence and prevent relapse in patients suffering from schizophrenia. Beyond clinical efficacy, growing attention is being paid to their potential impact on brain structure. This systematic review aims to synthesize available evidence from neuroimaging studies investigating the effects of SGA-LAIs on grey matter (GM) and white matter (WM) in individuals with schizophrenia. **Methods:** A systematic search of PubMed/MEDLINE, Scopus and the Cochrane Central Register of Controlled Trials was run from database inception to 13 August 2026, supplemented by Google Scholar, trial registries, and backward and forward citation tracking of the included studies. Eligible studies were original reports assessing the effects of SGA-LAIs on brain structure or brain function with magnetic resonance imaging (MRI) in patients with schizophrenia. Study selection followed the PRISMA 2020 statement, and data extraction focused on sample characteristics, treatment, imaging outcomes, and main findings. Risk of bias was appraised with RoB 2 and ROBINS-I. **Results:** Of 687 unique records screened, nine studies met the inclusion criteria. The evidence base is narrow: the nine studies correspond to only five independent samples, with six of them acquiring both a baseline and a follow-up scan and five providing a randomized comparison with oral antipsychotics, and the only molecules studied were risperidone LAI and paliperidone palmitate. Five randomized reports compared an SGA-LAI with oral antipsychotics and described better preservation of intracortical myelin and of cortical thickness with the long-acting formulation, most often in frontal regions, although the between-group difference reached statistical significance in only two of them and diffusion measures in one cohort moved in the opposite direction. Two cross-sectional functional MRI studies comparing risperidone LAI with first-generation depot medication described activation patterns closer to those of healthy controls in the LAI group, and one longitudinal resting-state study documented changes in regional homogeneity during paliperidone palmitate treatment. In some cases, structural or functional changes correlated with clinical or cognitive improvement. However, all included studies were judged to be at high or serious risk of bias, and the nine studies correspond to only five independent samples, so that the apparent convergence of findings partly reflects repeated analyses of the same participants; the limited number of studies and their methodological heterogeneity prevent firm generalizations. **Conclusions:** Preliminary and low-certainty evidence suggests that SGA-LAIs may be associated with better preservation of intracortical myelin and cortical thickness in schizophrenia, with convergent but weaker functional data and with diffusion findings that do not fit this reading. These results are hypothesis-generating and do not establish a neuroprotective effect. Larger and independent longitudinal studies with standardized imaging protocols and prespecified clinical endpoints are needed before any structural advantage of LAI formulations can be considered established.

## 1. Introduction

Schizophrenia is a severe psychiatric disorder characterized by a complex interplay of positive symptoms, negative symptoms, cognitive dysfunction, behavioral disturbances and social withdrawal [1]. Neuroimaging studies have consistently shown that it is associated with progressive brain changes, including reductions in grey matter (GM) volume and disruptions in white matter (WM) integrity [2,3], which are believed to contribute to functional impairment and may evolve over the course of the illness [4].

Antipsychotic drugs remain the cornerstone of treatment [5], but their long-term effects on brain structure are debated. Longitudinal studies have reported associations between cumulative antipsychotic exposure and GM volume reductions, particularly in frontal and total brain volume [6,7,8], whereas other work suggests that second-generation antipsychotics may exert neuroprotective effects or be associated with volume increases in specific regions such as the pallidum [9,10,11]. It has been hypothesized that continuous therapeutic exposure, as opposed to intermittent treatment, plays a critical role in shaping these trajectories [8].

Long-acting injectable (LAI) formulations were developed to address poor adherence, a major contributor to relapse and rehospitalization [12,13]: in a multicentre cohort followed for twelve months after a first episode of psychosis, patients discharged on an oral antipsychotic were readmitted more frequently than those discharged on an LAI [14]. Long regarded as a last resort, and at times stigmatized as a coercive tool, LAIs have been progressively reconsidered by clinicians and guidelines, which now advocate earlier and wider use, including at the first episode and in diagnoses other than schizophrenia [15,16,17,18,19,20,21,22,23]; early intervention services are the setting in which this shift is most consequential, although recommended models of care and pharmacological guidance remain heterogeneous across guidelines [24]. Second-generation antipsychotic (SGA) LAIs also offer more stable plasma drug levels and favourable tolerability [25,26].

Against this background, it has been proposed that the more continuous receptor occupancy achieved with these formulations may translate into a measurable effect on brain structure [27], and preservation of structural integrity has in turn been associated with better long-term functional outcomes [28,29]. Bartzokis and colleagues, for example, reported that risperidone LAI preserved frontal lobe WM volume compared with oral administration [30,31].

The evidence behind this claim, however, is thinner than its circulation in the clinical literature suggests: studies are few, samples are small, imaging protocols and follow-up durations are heterogeneous, and direct comparisons between formulations are rare. This is precisely why a systematic appraisal is needed. Mapping what has actually been measured, in whom, with which technique and with what degree of certainty, tells clinicians how much weight the structural argument can bear when an injectable formulation is proposed, and tells investigators which questions remain genuinely open. We therefore systematically reviewed neuroimaging studies assessing GM, WM, intracortical microstructure and brain function in adults with schizophrenia treated with SGA-LAIs, with particular attention to comparisons with oral formulations.

## 2. Materials and Methods

### 2.1. Protocol and Registration

This systematic review was conducted and reported according to the Preferred Reporting Items for Systematic Reviews and Meta-Analyses (PRISMA) 2020 statement; the completed PRISMA 2020 checklist is provided as Supplementary Table S4. The protocol was not registered in PROSPERO; this is acknowledged among the limitations of the review.

### 2.2. Eligibility Criteria

Studies were included if they met the following criteria:

Population: Adults (≥18 years) diagnosed with schizophrenia according to DSM-IV, DSM-5, or ICD-10/11 criteria.

Intervention: Treatment with second-generation long-acting injectable (LAI) antipsychotics (e.g., risperidone LAI, paliperidone palmitate, aripiprazole LAI, olanzapine pamoate).

Comparator: Oral antipsychotics, first-generation long-acting injectable (depot) antipsychotics, healthy controls, or within-subject change over time.

Outcomes: Changes in, or between-group differences in, brain structure or brain function assessed with magnetic resonance imaging, including structural MRI (sMRI), diffusion tensor imaging (DTI) and resting-state or task-based functional MRI (fMRI). Other imaging modalities, such as positron emission tomography, single-photon emission computed tomography and magnetic resonance spectroscopy, were not considered.

Study types: Longitudinal observational studies, cross-sectional comparative studies, and randomized or non-randomized clinical trials.

Language: Only studies published in English were considered.

Exclusion criteria:

Studies investigating only clinical outcomes without any neuroimaging data.

Studies evaluating exclusively first-generation LAI antipsychotics.

Studies in which neuroimaging results were not reported separately for the second-generation LAI arm.

Case reports, case series, narrative reviews, editorials, letters, or conference abstracts.

Animal studies.

### 2.3. Information Sources and Search Strategy

A comprehensive literature search was conducted in PubMed/MEDLINE, Scopus and the Cochrane Central Register of Controlled Trials, from database inception to 13 August 2026, with no lower date limit. The search combined three concept blocks with the Boolean operator AND: population (schizophrenia, schizoaffective disorder, psychosis, first-episode psychosis), intervention (generic terms for long-acting injectable and depot antipsychotic formulations together with the names of the individual injectable formulations and their brand names) and outcome (structural and functional neuroimaging terms, including magnetic resonance imaging, diffusion tensor imaging, cortical thickness, grey and white matter, intracortical myelin and resting-state measures). Within each block, free-text terms searched in the title and abstract were combined with the corresponding controlled vocabulary terms. Only reports published in English were considered. The complete search strings for each database, with the date of execution and the number of records retrieved, are reported in Supplementary Table S3. ClinicalTrials.gov and the WHO ICTRP were searched for registered trials with a neuroimaging component; backward and forward citation tracking was performed on all included studies, and Google Scholar was searched as a supplementary source using three predefined queries, with all retrieved records being screened.

### 2.4. Study Selection

Two independent reviewers screened the titles and abstracts of all identified records against the criteria listed above. Screening was assisted by an automated pre-classification generated with a general-purpose large language model (Claude, Anthropic), which was prompted with the eligibility criteria and returned, for each of the 687 records, a proposed decision (include, retrieve full text, or exclude) and a one-line reason. The tool was used as a first-pass triage and not as a decision rule: every record proposed for inclusion or for full-text retrieval (*n* = 24) was examined in full by both reviewers, and a random sample of 66 of the 663 records proposed for exclusion (10%, drawn with a fixed random seed) was independently re-screened against the eligibility criteria, with complete agreement with the automated proposal in all 66 cases. All final decisions were made by the two reviewers. Full-text articles were then retrieved and assessed for eligibility, and disagreements were resolved by discussion or by consulting a third reviewer.

### 2.5. Data Extraction

Data were independently extracted by two reviewers, with discrepancies resolved through discussion. The extracted information included: study characteristics (author, year, country, study design); sample characteristics (sample size, mean age, sex distribution, illness duration); type and dosage of LAI antipsychotic; duration of follow-up; neuroimaging techniques used; brain regions assessed; main findings regarding brain structure changes.

### 2.6. Risk of Bias Assessment

The risk of bias was assessed using two different tools, depending on the study design. For the four non-randomized studies [32,33,34,35], two longitudinal and two cross-sectional, we employed the Risk of Bias In Non-randomised Studies of Interventions (ROBINS-I) tool [36], which evaluates bias across seven domains. For the five randomized reports [30,31,37,38,39], which derive from two randomized trials, we used the Cochrane Risk of Bias 2 (RoB 2) tool [40], which assesses five domains specific to randomized trials. Each report was appraised separately, because the outcomes and the analysed subsamples differ between reports of the same trial. Two reviewers independently performed the assessments, and discrepancies were resolved through discussion. Judgments are reported in Section 3.2 and, at domain level, in Supplementary Tables S1 and S2.

### 2.7. Data Synthesis

Due to the expected variability in neuroimaging protocols, sample characteristics, and reported outcomes, a narrative synthesis was performed. No meta-analysis was attempted. Results were organized based on brain structure changes (grey matter vs. white matter) and, where available, differentiated according to the specific LAI antipsychotic used.

## 3. Results

### 3.1. Study Selection and Study Characteristics

The search of databases and registers retrieved 378 records (PubMed 55, Scopus 125, CENTRAL 80, Google Scholar 118), of which 62 were duplicates, leaving 316 records to be screened. Backward and forward citation tracking of the included studies retrieved a further 419 records, of which 48 had already been identified, leaving 371 additional records, so that 687 unique records were screened on title and abstract. Twenty-four reports were sought for retrieval and assessed in full text, of which 15 were excluded (imaging modality not eligible, *n* = 3; first-generation LAI only, *n* = 4; imaging results not reported separately for the second-generation LAI arm, *n* = 4; no LAI treatment or no comparison by formulation, *n* = 3; not peer-reviewed, *n* = 1), leaving nine studies, reported in ten records, included in the review (Figure 1). The characteristics of the included studies are summarized in Table 1 and Table 2.

Seven studies employed longitudinal neuroimaging designs and two were cross-sectional. All investigated changes in, or between-group differences in, white matter (WM), gray matter (GM), cortical microstructure or brain function in patients with schizophrenia treated with SGA-LAIs. Sample sizes ranged from 7 to 71 patients, and follow-up durations in the longitudinal studies varied between 13 weeks and 9 months. All included studies investigated risperidone LAI or paliperidone palmitate; no eligible study was retrieved for aripiprazole LAI or for olanzapine pamoate. Five studies compared an SGA-LAI with oral antipsychotics [30,31,37,38,39], two compared risperidone LAI with first-generation depot medication [34,35], one compared patients with healthy controls before and after treatment [33], and one had no comparison group [32]. Structural MRI, diffusion tensor imaging (DTI), cortical thickness and gray–white matter contrast analyses, and resting-state and task-based functional MRI were used across the included studies. Of note, the number of independent samples is considerably smaller than the number of included studies. Three reports [37,38,39] derive from the same randomized multicentre programme (the DREaM study), one of them having subsequently been the object of a corrigendum that added an omitted acknowledgment without modifying any reported result [39]. The two reports by Bartzokis and colleagues [30,31] are two analyses of a single randomized trial (NCT00330551), the second being restricted to the 22 of the 24 participants whose proton density images were usable. The two cross-sectional reports [34,35] describe the same patients: age, illness duration, PANSS and Global Assessment of Functioning values are identical in the two papers, and only the healthy control group differs, comprising 8 subjects in the earlier report and 16 in the later one. The nine included studies therefore correspond to five independent samples.

### 3.2. Risk of Bias

No study was rated at low overall risk of bias, and the domain-level judgments with their justifications are reported in Supplementary Tables S1 and S2.

All five randomized reports, which correspond to two trials, were at high overall risk of bias, driven in every case by missing outcome data: attrition between baseline and the final scan reached 33% in the trial of risperidone formulations [30,31] and was both substantial and differential between arms in DREaM, where 60.9% of the participants assigned to the LAI and 81.3% of those assigned to oral treatment completed the imaging assessments [39]. Concerns also arose for the randomization process, since allocation concealment is not described and the analysed samples were defined after randomization, and for selection of the reported result, which was rated high in the two supplementary analyses of the DREaM imaging data [37,38]. Measurement of the outcome was at low risk throughout, because the imaging measures are objective and the raters were blinded to allocation (Figure 2).

The four non-randomized studies were all at serious overall risk of bias with ROBINS-I, in every case because of confounding: the single-arm study has no comparison group [32], most patients in the open-label longitudinal study had prior antipsychotic exposure and dropout was related to tolerability and response [33], and in the two cross-sectional studies allocation was made by the treating clinicians, all patients in the LAI group had previously received conventional depots, and no baseline assessment is available [34,35] (Figure 3).

### 3.3. White Matter Outcomes

Five studies reported outcomes related to white matter. Bartzokis and colleagues observed that frontal white matter volume remained stable with risperidone LAI and declined significantly with oral risperidone, with a significant differential treatment effect [30], and that frontal intracortical myelin increased significantly within the LAI group but only at a trend level when the two treatment arms were compared directly [31]. Tishler and colleagues [39] showed that treatment with paliperidone palmitate LAI maintained intracortical myelin volume in the frontal lobe, whereas a significant decline was observed in patients receiving oral antipsychotics, the between-group difference again not reaching significance. Wang and colleagues [37] reported that intracortical myelin decreased significantly and gray–white matter contrast increased during oral treatment, whereas neither measure changed significantly under paliperidone palmitate. Diffusion measures point in a different direction: in the same cohort [38], the LAI group showed larger reductions in fractional anisotropy and larger increases in mean diffusivity than the oral group, changes that would conventionally be read as microstructural deterioration, although in three of the identified clusters the reduction in fractional anisotropy was positively associated with cognitive improvement.

### 3.4. Gray Matter Outcomes

Gray matter outcomes were reported in four studies. Altamura and colleagues [32] suggested that greater baseline GM volumes could predict better clinical response to paliperidone palmitate LAI over 24 weeks. Bartzokis and colleagues [30] observed an increase in GM volume in the oral risperidone group and a non-significant decrease in the LAI group; because total frontal lobe volume did not change, the authors attributed this reciprocal pattern to a myelination-driven shift in the gray–white matter boundary and not to a difference in GM tissue loss. Wang and colleagues [37] found that paliperidone palmitate LAI attenuated cortical thinning relative to oral treatments. Bai and colleagues [33] primarily investigated functional changes (regional homogeneity) and did not provide direct GM volumetric measures.

### 3.5. Functional Outcomes

Four studies assessed brain function. Bai and colleagues [33] used resting-state fMRI and found that 13 weeks of paliperidone palmitate were followed by increased regional homogeneity in orbitofrontal cortex, subgenual anterior cingulate, precuneus and posterior cingulate, and by decreased regional homogeneity in superior temporal gyrus, thalamus and pre/postcentral gyrus, with baseline occipital values predicting the degree of symptomatic and functional improvement. The two cross-sectional studies compared patients receiving risperidone LAI with patients receiving first-generation depot medication and with healthy controls. During a working memory task [34], patients on risperidone LAI showed the load-dependent deactivation of the medial prefrontal cortex also seen in controls, whereas the first-generation depot group over-activated that region and under-activated the ventrolateral prefrontal cortex as memory load increased. During implicit processing of facial emotions [35], the group receiving risperidone LAI and the healthy control group showed greater activation of the left amygdala to intensely fearful faces, and of the right cerebellum to intensely happy faces, than the group receiving first-generation depot medication, which again over-activated the ventromedial prefrontal cortex. In both studies, the activation pattern of the LAI group was closer to that of healthy controls, but the cross-sectional design and the naturalistic allocation to treatment preclude any causal interpretation. Finally, within the DREaM cohort [38], nine months of paliperidone palmitate reduced the fronto-temporal and thalamo-temporal connectivity that was abnormally high at baseline and increased fronto-sensorimotor and thalamo-insular connectivity, whereas oral treatment increased fronto-temporal and thalamo-temporal connectivity; cognitive performance improved in both arms.

### 3.6. Clinical Correlations

Some studies [30,32] suggested associations between brain structural changes and clinical improvement, such as better response on clinical scales or improved cognitive performance. However, clinical correlations were inconsistently reported across studies, limiting definitive conclusions.

### 3.7. Comparison with Oral and Depot Formulations

Adherence in the oral comparator arms was documented in only one report, which rated it on a composite scale combining pill counts, patient report, plasma antipsychotic levels, psychiatrist judgment and electronic monitoring caps [31]; in the remaining studies, adherence in the oral arm was inferred from prescription records or was not reported, and no study used systematic plasma-level monitoring to verify it. Five studies [30,31,37,38,39] included direct comparisons between an SGA-LAI and oral formulations. On measures of myelination and cortical structure, all of them favoured the LAI formulation, although in three of the five the between-group difference did not reach statistical significance and was reported as a trend or as an effect size in the predicted direction [31,38,39]. On diffusion measures, the picture is not concordant, since the LAI arm showed larger reductions in fractional anisotropy and larger increases in mean diffusivity than the oral arm [38]. Two further studies [34,35] compared risperidone LAI with first-generation depot antipsychotics and found functional activation patterns closer to those of healthy controls in the LAI group, although in these studies the contrast concerns both the formulation and the drug class and cannot separate the two. These findings suggest a potential neuroprotective advantage of long-acting injectable formulations in patients with schizophrenia, which must, however, be weighed against the risk-of-bias profile of the evidence base, the shared origin of three of the reports and the probable sample overlap of the two cross-sectional studies.

## 4. Discussion

This systematic review highlights preliminary evidence suggesting that SGA-LAIs, particularly risperidone and paliperidone formulations, may exert beneficial effects on brain structure in patients with schizophrenia. Before these findings are interpreted, their basis should be kept in view: the nine included studies correspond to five independent samples, so that part of what reads as replication across the literature is in fact repeated analysis of the same participants, and the apparent convergence of the published results is correspondingly weaker than the number of reports suggests. Across the studies reviewed, SGA-LAI treatment was associated with greater preservation of WM integrity, attenuation of GM loss, and, in some cases, with maintenance of cortical microstructure, compared to oral antipsychotic treatment; two cross-sectional functional studies point in the same direction against a first-generation depot comparator, with a much weaker design.

### 4.1. White Matter Changes

Preservation of white matter was one of the most consistent findings. Bartzokis and colleagues [30,31] showed that patients receiving risperidone LAI exhibited either maintenance or increases in frontal WM volume and intracortical myelination compared to those receiving oral risperidone, although only the volumetric comparison reached statistical significance. More recently, the DREaM neuroimaging reports [37,38,39] found that paliperidone palmitate LAI was associated with reduced deterioration of intracortical myelin and of cortical thickness compared to oral antipsychotic treatment. The diffusion findings from the same cohort are less easily reconciled with this reading, since fractional anisotropy decreased and mean diffusivity increased more markedly under the LAI than under oral treatment [38]. Reductions in fractional anisotropy after antipsychotic treatment have been described repeatedly and have sometimes accompanied favourable clinical outcome, and in that study three clusters showed a positive association between the change in fractional anisotropy and cognitive improvement; taken at face value, however, the diffusion data do not support a simple account of better microstructural preservation under long-acting formulations, and this divergence within a single randomized cohort remains unresolved and is discussed further in Section 4.8. These results are otherwise consistent with earlier work indicating that antipsychotic exposure can modify the frontal myelination trajectory in the early phases of schizophrenia [31,41].

### 4.2. Grey Matter Changes

The impact of SGA-LAIs on GM structure was more heterogeneous, and the available measures are difficult to interpret in isolation. Altamura and colleagues [32] observed that higher baseline GM volumes were predictive of better clinical response to paliperidone palmitate LAI after 24 weeks, although no post-treatment scan was acquired and no direct measure of GM change is available. In the randomized comparison of risperidone formulations [30], GM volume increased in the oral arm while frontal WM volume declined, and the opposite pattern was observed in the LAI arm, with the total frontal lobe volume unchanged; this reciprocity indicates that the two compartments cannot be read independently when myelin-sensitive sequences are used, since progressive myelination of the deeper cortical layers moves the measured boundary between them. Wang and colleagues [37] indicated that paliperidone palmitate LAI attenuated cortical thinning relative to oral treatments. These findings suggest a potential protective effect on GM, although more studies with volumetric analyses are needed to confirm this trend.

Reduced volume loss has been reported with second-generation compared with first-generation antipsychotics [42], and animal work points to neurogenesis and protection against oxidative stress and oligodendrocyte loss as candidate mechanisms [43,44,45]. The meaning of volumetric change in psychosis nevertheless remains debated, since MRI-derived differences may partly reflect artefacts or epiphenomena and not tissue loss [46,47], including alterations of the neuro-glio-vascular microenvironment such as glymphatic clearance, which volumetric measures do not capture [48].

### 4.3. Intracortical Myelin and Cortical Microstructure

Intracortical myelin (ICM), located predominantly in the deeper cortical layers, contributes to neuronal synchrony and to the speed of neural transmission; its disruption appears early in schizophrenia and worsens over time [31,49,50,51,52,53], and antipsychotic treatment may act on the signaling pathways involved in myelin development and repair [31,41].

In a cross-sectional study examining frontal ICM in patients with schizophrenia receiving second-generation oral antipsychotics, an initial increase in ICM volume was found, followed by a decline after the first year of treatment [41]. This inverted U-shaped trajectory appears to mirror the typical temporal pattern of treatment adherence and of the clinical outcomes seen in the early phases of the illness. SGA-LAIs may therefore contribute to neuroprotection by better sustaining ICM and supporting global WM volume, particularly in younger individuals at disease onset. Among the molecular mechanisms proposed for the effect of second-generation antipsychotics on WM is the modulation of glycogen synthase kinase-3 (GSK-3) and Akt signaling, which regulate oligodendrocyte differentiation and myelination [54,55,56]. Consequently, the improved adherence associated with LAI administration might offer greater neuroprotective advantages through a more stable modulation of pro-myelinating signals compared with oral treatments.

### 4.4. Functional and Microstructural Changes

Bai and colleagues [33] used resting-state functional MRI and reported altered regional homogeneity (ReHo) after 13 weeks of paliperidone palmitate LAI treatment. While these results suggest potential functional reorganization, the absence of a direct comparison with oral formulations in this study limits strong conclusions.

Two cross-sectional studies from the same group compared patients maintained on risperidone LAI with patients maintained on first-generation depot antipsychotics [34,35]. During a parametric working memory task, the LAI group showed the load-dependent deactivation of medial prefrontal cortex observed in healthy controls, whereas the depot group over-recruited that region and failed to engage ventrolateral prefrontal cortex as load increased [34]; during implicit processing of intense facial emotions, the LAI group again resembled controls in amygdala and cerebellar responses, while the depot group over-activated ventromedial prefrontal cortex [35]. Taken together, these findings are consistent with a normalizing effect of a second-generation LAI on task-related activation, but they are also compatible with a class effect of second-generation over first-generation antipsychotics, or with the clinical characteristics that led the treating clinicians to prescribe one formulation instead of the other. Since patients were assessed at a single time point and all patients in the LAI group had previously received conventional depots, no inference about change over time is possible.

ReHo measures the similarity between the time series of a voxel and those of its immediate neighbours, and indexes local temporal synchronization of the blood-oxygen-level-dependent signal [57,58]; abnormal spontaneous activity has been repeatedly documented in schizophrenia [59,60], including altered regional activity in the putamen and prefrontal cortex in patients with auditory verbal hallucinations [61]. The findings of Bai and colleagues suggest that paliperidone palmitate may modify resting-state activity, possibly through the thalamo-orbitofrontal network, although the mechanism remains unclear.

Cortical thinning in frontal, temporal, parietal, cingulate and insular regions is reported in both chronic and early-stage schizophrenia [2,3,62] and has been linked to cumulative antipsychotic exposure [6,8,63]; disentangling disease-related from treatment-related change remains difficult, although greater thinning is generally associated with poorer outcome. Consistent with this, Wang and colleagues [37] observed widespread prefrontal, frontal and temporal thinning under oral treatment and more limited, right-lateralized reductions under paliperidone palmitate.

### 4.5. Clinical Correlations

Several studies attempted to correlate imaging findings with clinical outcomes. Altamura and colleagues [32] linked higher baseline GM volumes to better symptomatic response, and Bartzokis and colleagues [30] found that WM integrity was associated with better cognitive performance and faster reaction times. None of the included studies examined the domains that affect everyday functioning beyond positive and negative symptoms; in a multicentre real-world study, paliperidone palmitate was associated with improvement in such non-core features of schizophrenia [64], which suggests that the clinical endpoints paired with imaging outcomes may have been too narrow. Overall, clinical correlations were inconsistently reported, and direct cause-and-effect relationships between brain structural changes and symptom improvement remain speculative.

### 4.6. Possible Mechanisms of Neuroprotection

Several mechanisms may underlie the potential neuroprotective effects of SGA-LAIs. First, enhanced adherence and more stable plasma drug levels minimize the peaks and troughs that could destabilize brain circuits [25,65]; imaging data on dopamine D2 receptor occupancy show that long-acting formulations maintain a high and continuous receptor blockade, which persists well beyond the nominal dosing interval for several first-generation depots [66], providing the pharmacodynamic basis for this hypothesis. Second, second-generation antipsychotics may attenuate microglial activation and neuroinflammatory pathways [67,68]. Third, preclinical and clinical data suggest that risperidone and paliperidone can support oligodendrocyte function and myelin maintenance [31,38]. Finally, sustained D2 receptor occupancy without marked peaks may limit the synaptic remodeling associated with psychotic relapses [69]. Taken together, these mechanisms suggest that stable dopaminergic modulation may contribute to structural preservation in schizophrenia, although none of them has been directly tested in the studies included in this review.

### 4.7. Comparison with Other Long-Acting Antipsychotics

Although most of the included studies focused on risperidone and paliperidone, evidence on other long-acting formulations remains scarce and less consistent. In a first-episode cohort treated according to a fixed protocol with the first-generation LAI flupenthixol decanoate, in which adherence was therefore assured, cortical volume reductions over the first year were nevertheless greater in patients than in matched healthy controls [70], indicating that guaranteed antipsychotic exposure does not by itself prevent progressive structural change. A randomized double-blind trial in antipsychotic-naive patients treated with risperidone LAI or flupenthixol decanoate found no difference between arms on any MRI measure and pooled the two groups, which precluded its inclusion here [71]; its main finding, a reduction in ventral diencephalon volume correlated with weight gain and with a fall in HDL-cholesterol, points to a metabolic reading of some treatment-related volumetric changes that none of the included studies addressed. For other second-generation LAI molecules, no longitudinal neuroimaging comparison against oral treatment met our inclusion criteria. For aripiprazole once-monthly, the only published imaging evidence is a single-photon emission computed tomography study in first-episode patients, which reported improvements in right frontal and temporal perfusion and in several cognitive indices after three months, without a control group and with changes that did not survive correction for multiple comparisons [72]; this modality falls outside our eligibility criteria, and the result should be regarded as exploratory. Register searching identified two completed randomized studies with prespecified structural imaging endpoints for aripiprazole once-monthly: a phase 4 trial in which prefrontal white matter fractional anisotropy was the primary endpoint, with summary results posted in the register but no full publication [73], and the PRELAPSE trial, which listed brain volumes and white matter integrity among its secondary outcomes [74] and has so far reported only clinical results [75]. Neither could be included, so the published structural evidence concerns almost exclusively risperidone and paliperidone, and differences among molecules remain an open question.

### 4.8. What the Imaging Measures Capture

The studies included in this review combine measures that are often described together as indices of brain structure but that derive from different physical signals and are sensitive to different biological features, which is the main reason why their results cannot be read as a single quantity. Intracortical myelin was estimated by subtracting the tissue delineated on proton density images from that delineated on inversion recovery images, a contrast that depends on the high cholesterol and lipid content of myelin membranes and that therefore tracks the myelinated portion of the deeper cortical layers rather than cortical volume as such [41,49]. Cortical thickness and grey-white matter contrast are derived from T1-weighted images through surface reconstruction, and the contrast at the grey-white boundary is itself influenced by intracortical myelination, so that progressive myelination can move the measured boundary and produce reciprocal changes in the grey and white matter compartments without any loss or gain of tissue [53,64]. Diffusion measures derive from the displacement of water molecules and are shaped by axonal membranes and their packing density, by myelination, by fibre orientation and crossing within the voxel, and by the extracellular and free water compartments; fractional anisotropy and mean diffusivity are therefore summary indices of a heterogeneous microenvironment and not measures of white matter integrity in any direct sense [76,77]. Functional measures add a further layer of indirection, since the blood-oxygen-level-dependent signal reflects neurovascular coupling and is sensitive to vascular tone, physiological noise and the state of the participant during acquisition [58].

This explains, without resolving, the divergence observed within the DREaM cohort, where myelin-sensitive indices favoured the long-acting formulation while fractional anisotropy decreased and mean diffusivity increased more under the same treatment [37,38]. A reduction in fractional anisotropy may follow from a loss of axonal coherence, but also from an increase in extracellular or free water, from altered crossing-fibre configurations, or from changes in perivascular and interstitial fluid dynamics, which have been proposed as a further determinant of imaging signals in psychiatric disorders and are themselves commonly estimated from diffusion-weighted data [48,78]. Different combinations of these processes can move myelin-sensitive and diffusion-based indices in opposite directions in the same tissue, and the available data do not allow the alternatives to be distinguished. The practical consequence for this literature is twofold: mechanistic claims about neuroprotection cannot rest on any single modality, and future studies should report myelin-sensitive, diffusion-based and functional measures on the same participants, so that convergence or divergence becomes part of the result and not an artefact of the technique chosen.

### 4.9. Strengths and Limitations

Most of the included studies employed longitudinal designs and advanced neuroimaging methodologies, and five of them were randomized, which strengthens the internal validity of those comparisons; the search was rebuilt from scratch across three databases and complemented by citation tracking and a supplementary source, and all judgments are reported at the domain level. Several limitations must nevertheless be acknowledged. The evidence base is small and unevenly informative. Sample sizes ranged from 7 to 71 participants and follow-up durations from 13 weeks to 9 months, so that any effect on slowly evolving structural trajectories can only be partially captured; moreover, only six of the nine included studies acquired both a baseline and a follow-up scan, since one acquired imaging at baseline only and two were cross-sectional, and only five provided a randomized comparison with oral antipsychotics. All nine studies were judged to be at high or serious risk of bias, mainly because of missing outcome data in the randomized trials and of confounding and participant selection in the non-randomized ones, which lowers the certainty of the whole body of evidence. The nine included studies correspond to only five independent samples: three reports derive from the DREaM programme, two are separate analyses of a single randomized trial of risperidone formulations, and the two cross-sectional studies describe the same patients with different control groups. The evidence base is therefore weighted towards a small number of cohorts and two molecules, and the apparent convergence of findings partly reflects repeated analysis of the same participants, and not independent replication. The two cross-sectional studies compare an SGA-LAI with first-generation depot medication and therefore confound formulation with drug class, and their single time point makes any inference about structural or functional trajectories impossible. Imaging techniques, analytic pipelines and outcome definitions were heterogeneous, and clinical correlation analyses were inconsistently reported, which precluded any quantitative synthesis. Within the single cohort in which both were measured, myelin-sensitive and diffusion-based indices moved in opposite directions, which cautions against treating “white matter integrity” as a single construct across studies. Finally, the review protocol was not registered; the search was restricted to studies published in English, Embase, PsycINFO and Web of Science were not searched because of lack of institutional access, the grey literature was not searched, and no formal assessment of the certainty of evidence was performed. With so few studies, funnel plot asymmetry could not be assessed, but the risk of publication bias is not merely theoretical: at least two completed randomized trials with prespecified structural imaging endpoints for a different molecule remain unpublished in full [73,74], so the possibility that unreported results differ systematically from the published ones cannot be excluded. These factors prevent definitive conclusions and highlight the need for more robust studies.

### 4.10. Future Research Directions

Future studies should adopt standardized neuroimaging protocols, larger samples, and longer follow-up durations to clarify the impact of SGA-LAIs on brain structure, and should prespecify clinical and cognitive endpoints alongside imaging outcomes. Investigations comparing different LAI molecules, and independent replications outside the currently dominant cohorts, will be essential to establish whether the structural differences observed so far are clinically meaningful. Analytic approaches based on machine learning applied to functional imaging are being developed to derive reproducible individual-level markers in schizophrenia [79] and may help to move this field beyond group-level comparisons in small samples.

## 5. Conclusions

Evidence from a small and methodologically heterogeneous body of neuroimaging literature suggests that second-generation LAI antipsychotics may be associated with better preservation of white matter integrity and intracortical myelin, and with attenuated grey matter loss, compared with oral formulations in patients with schizophrenia; two cross-sectional functional studies additionally describe task-related activation patterns closer to those of healthy controls than in patients treated with first-generation depot medication. Given the small samples, the short follow-up durations, the fact that nine studies correspond to five independent samples, the divergence between myelin-sensitive and diffusion-based measures within the same cohort, the confounding between formulation and drug class in the cross-sectional comparisons and the high or serious risk of bias of all included studies, these findings should be regarded as hypothesis-generating, and not as established evidence of neuroprotection. These findings are hypothesis-generating and do not constitute evidence of neuroprotection: the direction of the structural signal is consistent, but the number of independent samples, the risk-of-bias profile and the divergence between imaging modalities place it well short of an established effect. Mapping this literature nonetheless has practical value. For clinicians, it defines how much weight the structural argument can bear in a conversation about treatment options, which at present is little; for investigators, it identifies what is missing, namely independent cohorts, molecules other than risperidone and paliperidone, and imaging endpoints prespecified alongside clinical and cognitive outcomes. Larger and independent longitudinal studies with standardized imaging protocols are needed before any neuroprotective advantage of LAI formulations can be considered established.

## Figures and Tables

**Figure 1:**
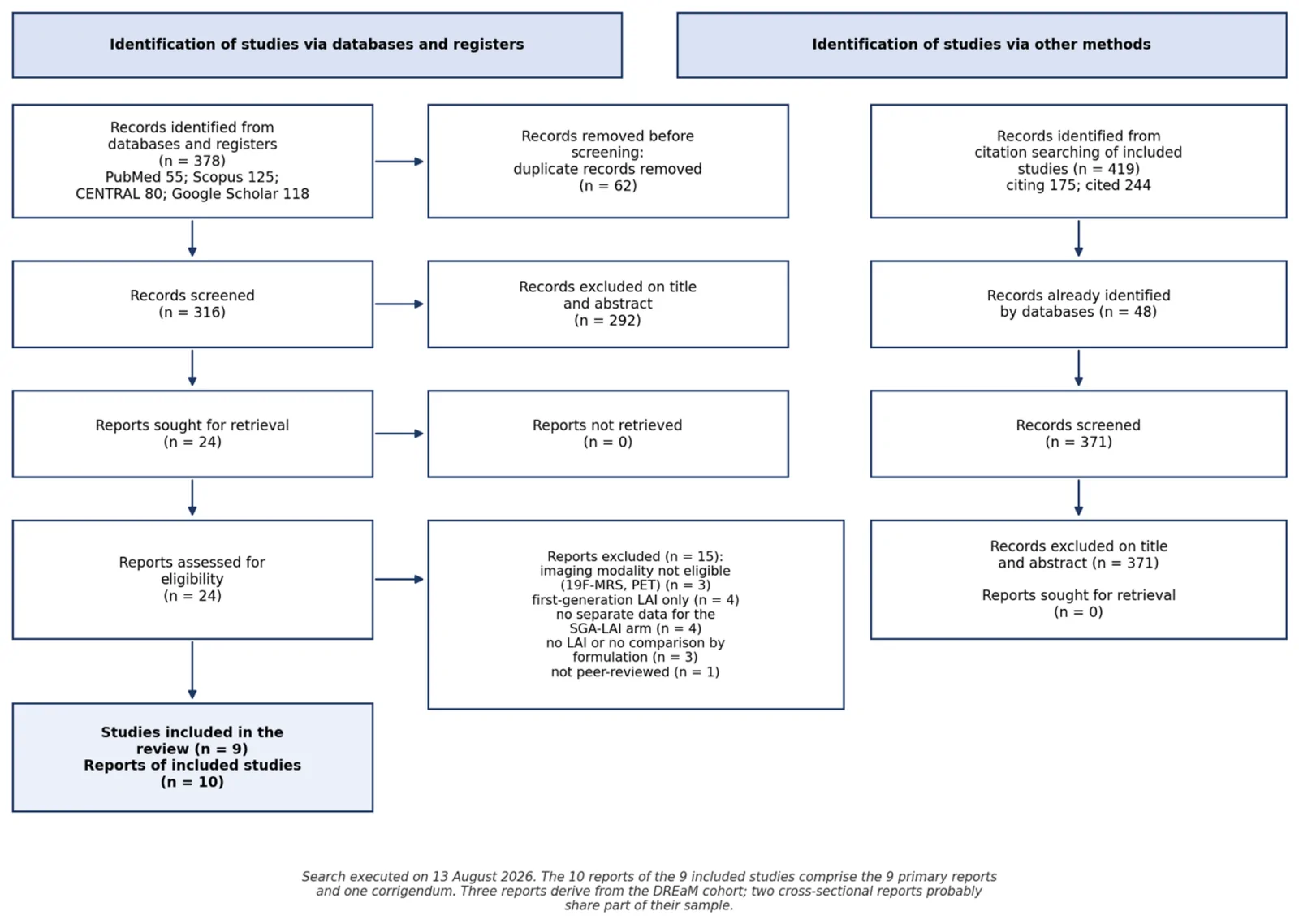
PRISMA 2020 flow diagram summarizing the study selection process.

**Figure 2:**
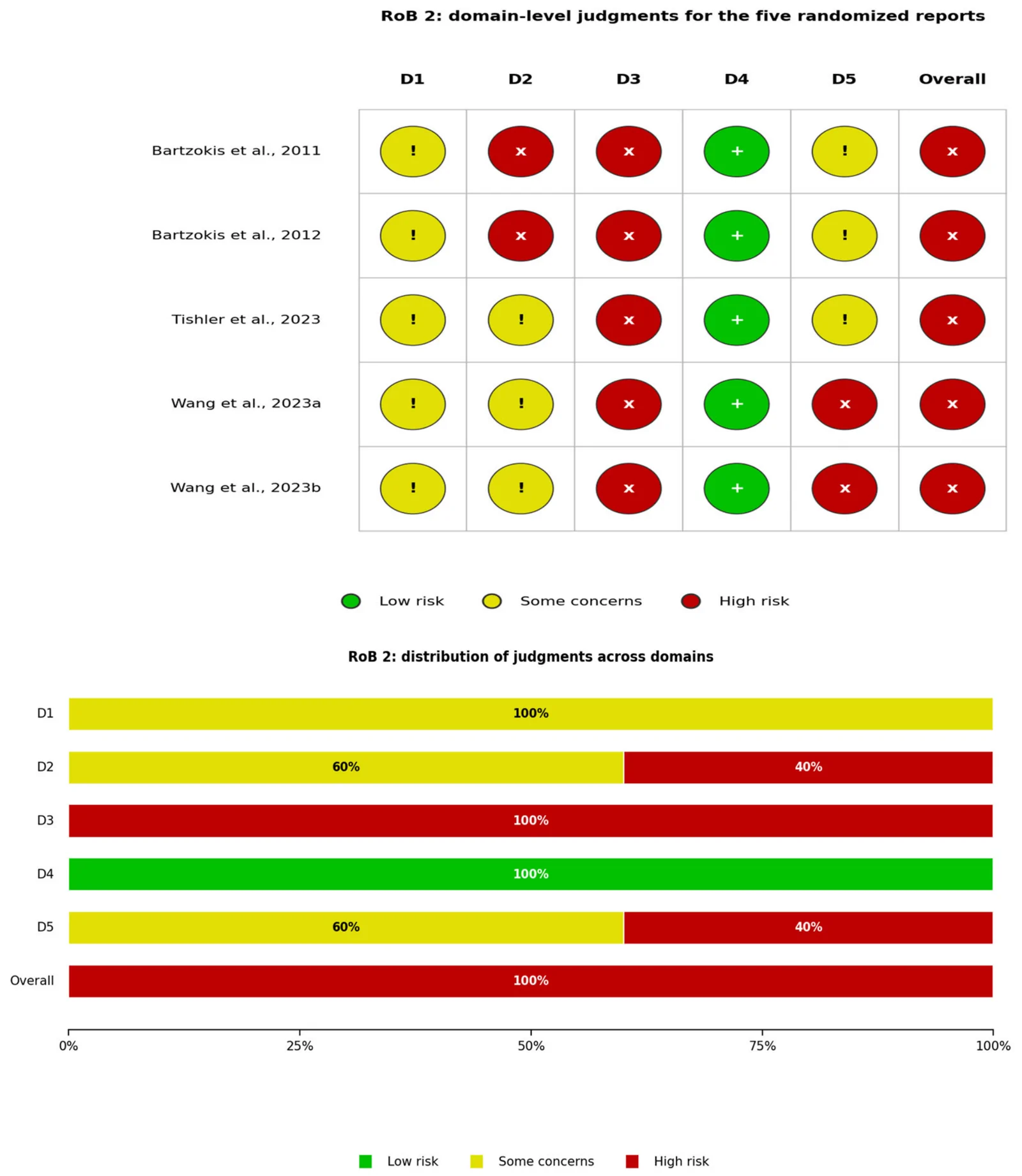
(**Upper Panel**) RoB 2 domain-level and overall judgments for the five randomized reports. D1, randomization process; D2, deviations from intended interventions; D3, missing outcome data; D4, measurement of the outcome; D5, selection of the reported result. (**Lower Panel**) Distribution of RoB 2 judgments across domains. 1. Bartzokis et al. (2011) [30]; 2. Bartzokis et al. (2012) [31]; 3. Tishler et al. (2023) [39]; 4. Wang et al. (2023a) [37]; 5. Wang et al. (2023b) [38].

**Figure 3:**
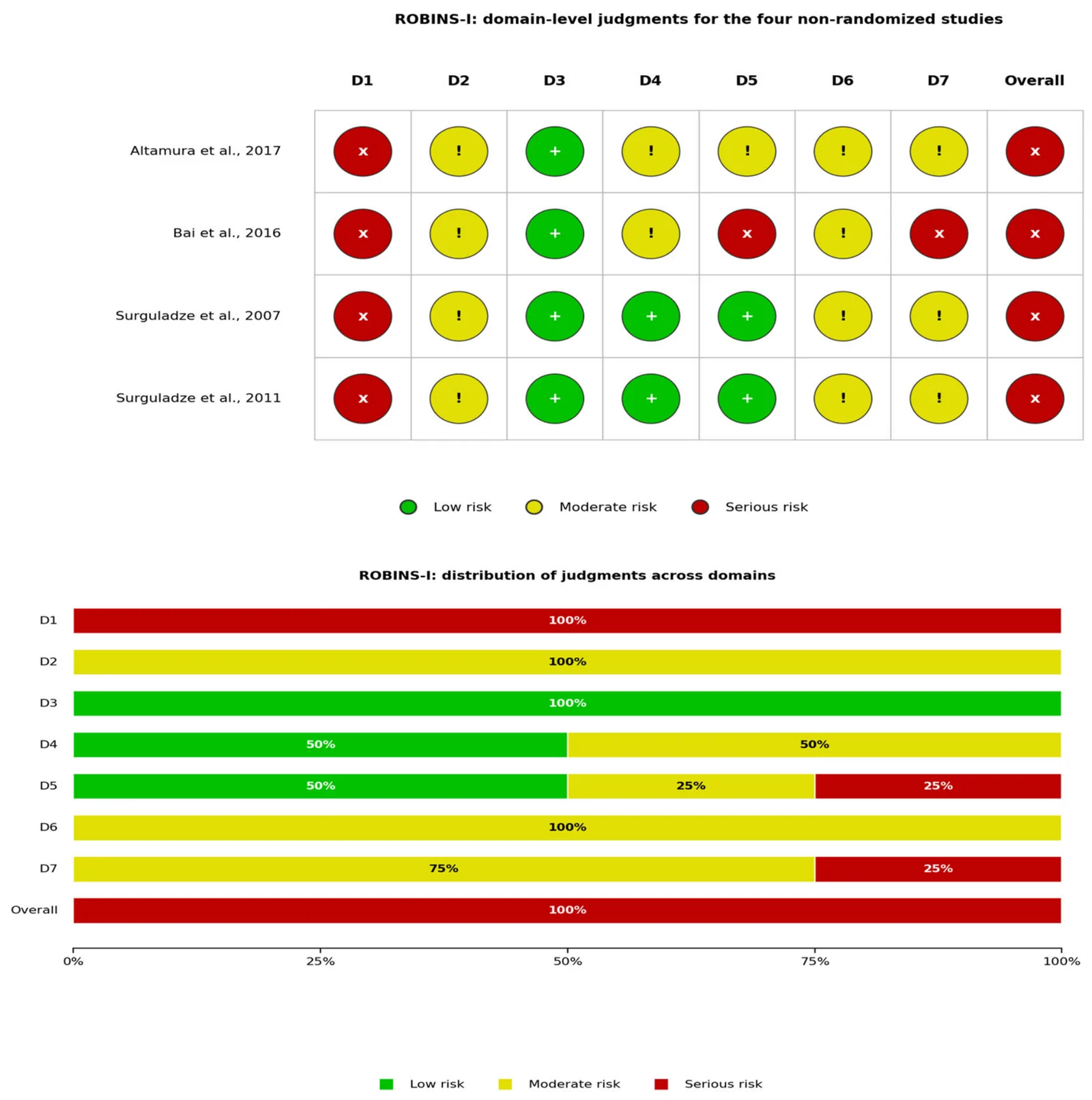
(**Upper Panel**) ROBINS-I domain-level and overall judgments for the four non-randomized studies. D1, confounding; D2, selection of participants; D3, classification of interventions; D4, deviations from intended interventions; D5, missing data; D6, measurement of outcomes; D7, selection of the reported result. (**Lower Panel**) Distribution of ROBINS-I judgments across domains. 1. Altamura et al. (2017) [32]; 2. Bai et al. (2016) [33]; 3. Surguladze et al. (2007) [34]; 4. Surguladze et al. (2011) [35].

**Table 1:** Sample and treatment characteristics of the included studies.

Study, Country	Design and Comparator	Sample: n, Mean Age (SD), Sex	Illness Duration	LAI and Dose	Follow-Up
Altamura et al. (2017) [32]; Italy	Prospective observational, single arm; no comparison group	7 patients (5 schizophrenia, 2 substance-induced psychosis); 31.0 (11.5) years; 6 M/1 F	8.3 (7.0) years; duration of untreated illness 30.1 (32.7) months	Paliperidone palmitate 150 mg on day 1 and 100 mg on day 8, then 50–150 mg every 28 days; mean maintenance dose 114.2 (24.4) mg eq.	24 weeks (MRI acquired at baseline only)
Bai et al. (2016) [33]; China	Longitudinal, non- randomized, open label; matched healthy controls	17 patients and 17 controls; patients 26.0 (5.5), controls 28.7 (6.0) years; 14 M/3 F in both groups	40.3 (38.0) months (range 7–120)	Paliperidone palmitate 150 mg on day 1 and 100 mg on day 8, then flexible 75, 100 or 150 mg monthly	13 weeks
Bartzokis et al. (2011) [30]; USA (UCLA Aftercare Research Program)	Randomized, open-label controlled trial (NCT00330551); oral risperidone and healthy controls	24 patients (11 risperidone LAI, 13 oral) and 14 controls; patients 24.6, controls 24.4 years, range 18–33; patients 18 M/6 F, controls 8 M/6 F	Median 6 months (SD 5.9) since onset of the first episode; mean antipsychotic exposure at baseline scan 7.2 months (range 4.3–14.2)	Risperidone LAI, mean 26.4 (4.2) mg every 2 weeks, modal dose 25 mg, range 12.5–37.5; oral risperidone, mean 2.9 (1.8) mg/day	6 months
Bartzokis et al. (2012) [31]; USA (same trial as Bartzokis et al. (2011) [30])	Randomized, open-label controlled trial; oral risperidone and healthy controls	22 patients (9 risperidone LAI, 13 oral) and 12 controls; LAI 25.1 (4.9), oral 23.5 (4.6), controls 24.2 years; LAI 7 M/2 F, oral 9 M/4 F, controls 8 M/4 F	Median 6 months (SD 5.9) since first episode onset; mean antipsychotic exposure at baseline 7.35 months; age at onset 24.2 (5.0) years (risperidone LAI) and 23.0 (4.5) years (oral).	Risperidone LAI, mean 26.4 (4.2) mg every 2 weeks; oral risperidone, mean 2.9 (1.8) mg/day	6 months (mean interval between scans 7.8 and 7.4 months)
Tishler et al. (2023) [39] (DREaM); USA and Mexico (UCLA, University of Pennsylvania, INNN Mexico City)	Randomized, open-label, delayed-start multicentre trial, 2:1; oral antipsychotics and healthy controls	71 patients (23 SGA-LAI, 48 oral) and 64 controls; 22.9 (3.7) LAI, 22.7 (3.9) oral, 22.9 (3.0) controls; 18 M/5 F SGA-LAI, 40 M/8 F oral	Time since diagnosis 0.4 years in both arms; time since first psychotic episode 0.9 (0.7) years SGA-LAI and 0.7 (0.5) years oral	Paliperidone palmitate once- monthly for at least 4 months, then 3-month formulation; flexible dose, not reported numerically	9 months (baseline, day 92, day 260)
Wang et al. (2023a) [37] (DREaM); same sites	Randomized multicentre trial, 2:1; oral antipsychotics and healthy controls	68 patients (21 SGA-LAI, 47 oral) and 63 controls; patients 22.6 (3.7) years, controls 23.0 (2.9); patients 57 M/11 F, controls 46 M/17 F	12.8 (7.4) months since psychosis onset	Paliperidone palmitate, 1-month formulation then 3-month formulation in 15 of 21; chlorpromazine equivalents 296.1 (149.8) mg/day in the SGA-LAI arm	9 months
Wang et al. (2023b) [38] (DREaM); same cohort as Wang et al. (2023a) [37]	Randomized multicentre trial, 2:1; oral antipsychotics and healthy controls	68 patients (21 SGA-LAI, 47 oral) and 63 controls; patients 22.6 (3.7) years; patients 57 M/11 F	12.9 (7.4) months since psychosis onset	Paliperidone palmitate, 1-month formulation for at least 4 months then 3-month formulation; chlorpromazine equivalents 296.1 (149.8) mg/day in the SGA-LAI arm	9 months
Surguladze et al. (2007) [34]; United Kingdom (London)	Cross-sectional, partly randomized allocation, with a small nested longitudinal sub-study; first-generation depot antipsychotics and healthy controls	16 patients on risperidone LAI, 16 on depot, 8 controls; 42.6 (11.7), 43.7 (9.4) and 32.8 (4.7) years; 7 M/9 F risperidone LAI, 10 M/6 F depot, 4 M/4 F controls.	15.3 (7.5) years risperidone LAI; 18.6 (9.7) years first-generation depot	Risperidone LAI 25–37.5 mg every 2 weeks for at least 3 months, approximately 194 mg/day chlorpromazine equivalents; first-generation depots approximately 232 mg/day.	Single assessment; nested sub- study of 17 patients randomized to switch to LAI or continue depot, rescanned after 3–4 months
Surguladze et al. (2011) [35]; United Kingdom (London)	Cross-sectional, naturalistic allocation; first-generation depot antipsychotics and healthy controls	16 patients on risperidone LAI, 16 on depot, 16 controls; 42.6 (11.7), 43.7 (9.4) and 40.4 (12.8) years; 7 M/9 F risperidone LAI, 10 M/6 F dep	ot, 8 M/8 F controls 15.3 (7.5) years risperidone LAI; 18.6 (9.7) years first-generation depot	Risperidone LAI 25–37.5 mg every 2 weeks for at least 3 months; first-generation depots approximately 232	mg/day chlorpromazine equivalents. Single assessment

LAI = long-acting injectable; SD = standard deviation. Values are mean (SD) unless otherwise stated.

**Table 2:** Imaging methods and main findings of the included studies.

Study	Imaging Modality	Brain Outcomes Measured	Main Findings
Altamura et al. (2017) [32]	3 T structural MRI, voxel-based morphometry	Total GM volume at baseline	Higher baseline total GM volume was associated with greater symptomatic improvement after 24 weeks of paliperidone palmitate
Bai et al. (2016) [33]	1.5 T resting-state fMRI	Regional homogeneity (ReHo)	After 13 weeks, ReHo increased in orbitofrontal cortex, subgenual anterior cingulate, precuneus and posterior cingulate and decreased in superior temporal gyrus, thalamus and pre/postcentral gyrus; baseline occipital ReHo predicted the degree of symptom reduction
Bartzokis et al. (2011) [30]	Structural MRI, inversion recovery	Frontal lobe WM and GM volume; reaction times	Frontal WM volume remained stable with risperidone LAI and decreased significantly with oral risperidone, with a significant differential treatment effect; healthy controls were intermediate. GM volume increased in the oral group and decreased non-significantly in the LAI group, which the authors attribute to a myelination-driven shift in the GM/WM boundary and not to GM loss. Greater WM increase was associated with faster reaction times on working memory and set-shifting tasks
Bartzokis et al. (2012) [31]	Structural MRI, inversion recovery and proton density	Frontal lobe intracortical myelin	ICM increased significantly in the LAI group (*p* = 0.005) and non-significantly in the oral group (*p* = 0.39) relative to healthy controls; the between-group difference was at trend level only (*p* = 0.093, Cohen’s d = 0.81). Better-rated adherence was associated with larger ICM increases
Tishler et al. (2023) [39] (DREaM)	3 T structural MRI, inversion recovery minus proton density subtraction	Frontal lobe intracortical myelin volume	Frontal ICM was preserved with paliperidone palmitate and decreased significantly with oral antipsychotics; the between-group difference did not reach significance, with a medium effect size in the predicted direction
Wang et al. (2023a) [37] (DREaM)	3 T structural MRI (MPRAGE, proton density, inversion recovery)	Cortical thickness, gray–white matter contrast, frontal ICM	Oral treatment was followed by widespread cortical thinning, a significant ICM decrease and a GWC increase, whereas no significant change in ICM or GWC was observed in the LAI group
Wang et al. (2023b) [38] (DREaM)	DTI and resting-state fMRI	WM microstructure (FA, MD), functional and microstructural connectivity, cognition (MCCB)	Paliperidone palmitate reduced the abnormally high fronto-temporal and thalamo-temporal connectivity and increased fronto-sensorimotor and thalamo-insular connectivity, whereas oral treatment increased fronto-temporal and thalamo-temporal connectivity. On diffusion measures, the LAI group showed larger FA decreases and larger MD increases than the oral group, a direction opposite to that of preserved microstructure; the authors read these changes as possible reorganization and not as deterioration, noting that in three clusters FA change correlated positively with cognitive improvement
Surguladze et al. (2007) [34]	1.5 T task-based fMRI, n-back working memory task	Activation of the working memory network	Patients on risperidone LAI, like healthy controls, deactivated the ventromedial prefrontal cortex as memory load increased, whereas the depot group over-activated that region (*p* = 0.013 vs. LAI); the depot group also under-activated the ventrolateral prefrontal cortex and performed worse on the 3-back task than controls. The nested longitudinal sub-study was underpowered but showed changes in the same direction after switching to LAI
Surguladze et al. (2011) [35]	1.5 T event-related fMRI, facial emotion processing	Amygdala, ventromedial prefrontal cortex, temporal pole, cerebellum	The LAI and control groups showed greater activation of the left amygdala to intensely fearful faces and of the right cerebellum to intensely happy faces than the depot group, which over-activated the ventromedial prefrontal cortex

DTI = diffusion tensor imaging; FA = fractional anisotropy; fMRI = functional magnetic resonance imaging; GM = gray matter; GWC = gray–white matter contrast; ICM = intracortical myelin; LAI = long-acting injectable; MCCB = MATRICS Consensus Cognitive Battery; MD = mean diffusivity; MRI = magnetic resonance imaging; ReHo = regional homogeneity; WM = white matter.

## Data Availability

No new data were created or analyzed in this study. The screening and data extraction files generated during the review are available from the corresponding author upon reasonable request.

## Supplementary Materials

The following supporting information can be downloaded at: https://www.mdpi.com/article/10.3390/brainsci16101020/s1; Table S1: RoB 2 domain-level judgments for the five randomized reports, with justifications; Table S2: ROBINS-I domain-level judgments for the four non-randomized studies, with justifications; Table S3: Information sources, search strings as run, date of execution and number of records retrieved; Table S4: PRISMA 2020 checklist, based on the PRISMA 2020 statement [80].
