# Supplementary material for: Effects of Second-Generation Long-Acting Injectable Antipsychotics on Brain Structure in Schizophrenia: A Systematic Review of Neuroimaging Studies
Source: Brain Sci. 2026 Sep 25;16(10):1020. doi: 10.3390/brainsci16101020 (PMC13702106; doi:10.3390/brainsci16101020)
Supplement: Supplementary file 1 [file brainsci-16-01020-s001.zip › brainsci-4556893-supplementary.pdf]

Supplementary Materials

Table S1. RoB 2 domain-level judgments for the five randomized reports, with justifications.

Study	Domain	Judgment	Justification
Bartzokis et al. (2011) [30]	D1 Randomization process	Some concerns	Allocation by random number table, but allocation concealment not described; the analysed sample was defined after randomization by an a priori exposure-matching rule that excluded five participants; racial composition differed significantly between arms (p = 0.017)
	D2 Deviations from intended interventions	High	Open-label trial; participants switched to another antipsychotic before the endpoint were excluded from the analysis instead of being analysed as randomized
	D3 Missing outcome data	High	33% attrition between baseline and 6 months (9 lost to follow- up, 6 switched medication); no imputation and no analysis of the impact of missingness on the estimate
	D4 Measurement of the outcome	Low	Volumetric MRI outcome, objective; scans rated in random order by a single rater blinded to clinical and demographic characteristics
	D5 Selection of the reported result	Some concerns	No publicly available prespecified analysis plan for the imaging outcomes; adjustment for race was introduced because of the observed baseline imbalance
	Overall	High	Determined by missing outcome data and by the exclusion of participants who deviated from the assigned intervention
Bartzokis et al. (2012) [31]	D1 Randomization process	Some concerns	Same trial and same limitations as the 2011 report; the analysed sample is further reduced by exclusion of participants with artefacts on proton density images
	D2 Deviations from intended interventions	High	Open-label trial; per-protocol analysis, with exclusion of participants switched to another antipsychotic
	D3 Missing outcome data	High	33% attrition, plus additional exclusions for image quality; 22 of the 45 recruited participants contribute to the analysis
	D4 Measurement of the outcome	Low	Intracortical myelin measured on inversion recovery and proton density images by a rater blinded to clinical and demographic characteristics; intra-rater reliability reported
	D5 Selection of the reported result	Some concerns	The between-group comparison is reported as a trend (p = 0.093) alongside within-group tests and analyses based on median splits of both adherence and volume change, defined on the observed data
	Overall	High	Determined by missing outcome data and deviations from the intended interventions
Tishler et al. (2023) [39] (DREaM)	D1 Randomization process	Some concerns	Randomized 2:1 within a multicentre trial, but the imaging population is an optional subsample recruited at three of thirty-four sites; allocation concealment not described for the imaging component
	D2 Deviations from intended interventions	Some concerns	Open-label, flexible dose, with supplementation by another antipsychotic permitted; discontinuation was more frequent in the LAI arm (39.1% vs. 18.8%)
	D3 Missing outcome data	High	Only 14 of 23 participants in the LAI arm (60.9%) and 39 of 48 in the oral arm (81.3%) completed the Part II imaging assessments, with differential attrition between arms
	D4 Measurement of the outcome	Low	Imaging raters at the central site were blind to treatment assignment and to clinical and demographic characteristics; centralized scoring
	D5 Selection of the reported result	Some concerns	Imaging was a secondary endpoint of a trial whose primary endpoint was not met, and the authors describe the imaging evaluation as hypothesis-generating; results are reported from both ANCOVA and mixed-model analyses
	Overall	High	Determined by missing outcome data
Wang et al. (2023a) [37] (DREaM)	D1 Randomization process	Some concerns	As for the parent trial: imaging subsample from three sites, allocation concealment not described
	D2 Deviations from intended interventions	Some concerns	Open-label with flexible dosing; chlorpromazine equivalent dose differed between arms at baseline (p = 0.087) and was added as a covariate in supplementary analyses

Study	Domain	Judgment	Justification
	D3 Missing outcome data	High	14 participants in the oral arm and 9 in the LAI arm were lost to follow-up, leaving 12 of 21 in the LAI arm at nine months
	D4 Measurement of the outcome	Low	Objective imaging outcomes processed with standard automated pipelines; intracortical myelin scored by raters blind to allocation
	D5 Selection of the reported result	High	The authors state that these are supplementary analyses, going beyond the planned imaging analyses of the trial; whole-brain vertex-wise and region-of-interest analyses are reported selectively, with regions of interest defined on the observed data
	Overall	High	Determined by missing outcome data and by selection of the reported result
Wang et al. (2023b) [38] (DREaM)	D1 Randomization process	Some concerns	As for the parent trial and the companion report
	D2 Deviations from intended interventions	Some concerns	Open-label with flexible dosing; the authors note that dose differences between arms may affect connectivity measures
	D3 Missing outcome data	High	Imaging at nine months available for 45 of the 68 participants scanned at baseline
	D4 Measurement of the outcome	Low	Diffusion and functional measures derived with standard automated pipelines, with quality control performed in real time
	D5 Selection of the reported result	High	Exploratory analyses across many regions of interest and white matter tracts, with a voxel-wise threshold of $p < 0.05$ uncorrected before cluster-level correction, and no prespecified imaging analysis plan
	Overall	High	Determined by missing outcome data and by selection of the reported result

D1, randomization process; D2, deviations from intended interventions; D3, missing outcome data; D4, measurement of the outcome; D5, selection of the reported result. The five reports derive from two randomized trials: Bartzokis et al. (2011) [30] and (2012) [31] report two analyses of the same trial (NCT00330551), and Tishler et al. (2023) [39] and Wang et al. (2023a) [37] and (2023b) [38] report three analyses of the imaging component of the DREaM trial (NCT02431702). Each report was appraised separately because the outcomes and the analysed subsamples differ.

Table S2. ROBINS-I domain-level judgments for the four non-randomized studies, with justifications.

Study	Domain	Judgment	Justification
Altamura et al. (2017) [32]	D1 Confounding	Serious	Single-arm design with no comparison group; the association between baseline grey matter and clinical response is adjusted only for intracranial volume; three patients received concomitant psychotropic medication, and two had a substance- induced psychosis
	D2 Selection of participants	Moderate	Consecutive recruitment of acutely admitted patients is implied but not documented; no data on eligible non-participants
	D3 Classification of interventions	Low	Formulation and dose administered by healthcare professionals on a fixed schedule and documented prospectively
	D4 Deviations from intended interventions	Moderate	Other therapy permitted in case of necessity, without reporting of how often this occurred
	D5 Missing data	Moderate	All seven patients are reported at 24 weeks, but no flow of eligible and excluded patients is given
	D6 Measurement of outcomes	Moderate	Imaging measure objective, but acquired only at baseline, and the clinical outcome was rated without blinding
	D7 Selection of the reported result	Moderate	Exploratory analysis with post hoc repetition after exclusion of the single female patient; no prespecified analysis plan
	Overall	Serious	Determined by confounding: without a comparison group, the observed changes cannot be attributed to the long-acting formulation
Bai et al. (2016) [33]	D1 Confounding	Serious	Eleven of the seventeen analysed patients had previously received antipsychotics, and only six were antipsychotic-naive; no adjustment for prior exposure, and the healthy control group received no treatment, so that treatment and illness effects cannot be separated
	D2 Selection of participants	Moderate	Consecutive inpatients and outpatients over a defined period, with criteria reported, but no data on eligible non- participants
	D3 Classification of interventions	Low	Injections administered and documented within the study, with a defined titration schedule
	D4 Deviations from intended interventions	Moderate	Open design with flexible dosing (75, 100 or 150 mg) decided by clinical judgment, and no report of concomitant medication
	D5 Missing data	Serious	Nine of the twenty-six enrolled patients did not contribute to the analysis, with dropout explicitly related to side effects, dissatisfaction with efficacy or symptom severity, so that missingness is likely to depend on the outcome
	D6 Measurement of outcomes	Moderate	Imaging measure objective, but symptom ratings were not blinded, and the control group was scanned without treatment
	D7 Selection of the reported result	Serious	Numerous exploratory whole-brain and correlational analyses across symptom and functioning scales, with thresholds set by Monte Carlo simulation and no prespecified analysis plan
	Overall	Serious	Determined by confounding, missing data and selection of the reported result
Surguladze et al. (2007) [34]	D1 Confounding	Serious	Allocation to risperidone LAI or to conventional depot was made by the treating clinicians in almost half of the sample, so that confounding by indication cannot be excluded; all patients in the LAI group had previously received conventional depots; healthy controls were about ten years younger than both patient groups; no baseline assessment before starting the current formulation. The judgment is serious because exposure and its duration are documented from clinical records and the two patient groups are closely matched on symptoms, illness duration and quality of life, so that the comparison, although confounded, remains informative
	D2 Selection of participants	Moderate	Recruitment from a geographically defined catchment area, restricted to clinically stable patients; no data on eligible non-participants
	D3 Classification of interventions	Low	Formulation, dose and duration of maintenance treatment documented from clinical records, with a minimum of three months of stable treatment
	D4 Deviations from intended interventions	Low	Single assessment, so that no deviation can occur between exposure and outcome measurement
	D5 Missing data	Low	Complete data for the cross-sectional analysis of the 32 patients and 8 controls

Study	Domain	Judgment	Justification
	D6 Measurement of outcomes	Moderate	BOLD measures are objective, but blinding of the analysts to treatment group is not stated
	D7 Selection of the reported result	Moderate	The region-of-interest analysis was defined from the trends observed in the control group, and the nested longitudinal sub-study is reported without correction for its multiple comparisons
	Overall	Serious	Determined by the confounding domain
Surguladze et al. (2011) [35]	D1 Confounding	Serious	Naturalistic allocation decided by the responsible clinicians; all patients in the LAI group had previously received conventional depots; no baseline assessment; a trend towards better global functioning in the LAI group ($p = 0.06$) suggests some non-comparability of the groups. As in the companion report, exposure is documented, and the groups are matched on symptoms, illness duration and quality of life, which keeps the comparison informative
	D2 Selection of participants	Moderate	Same catchment-area recruitment as the 2007 report, with no data on non-participants
	D3 Classification of interventions	Low	Formulation and dose documented from clinical records, with at least three months of stable treatment
	D4 Deviations from intended interventions	Low	Single assessment
	D5 Missing data	Low	Complete data for the analysed sample of 32 patients and 16 controls
	D6 Measurement of outcomes	Moderate	Objective BOLD measures, but no statement of blinding of the analysts to treatment group
	D7 Selection of the reported result	Moderate	Whole-brain analysis complemented by a region-of-interest analysis of the amygdala defined a posteriori; no prespecified analysis plan
	Overall	Serious	Determined by the confounding domain

D1, confounding; D2, selection of participants; D3, classification of interventions; D4, deviations from intended interventions; D5, missing data; D6, measurement of outcomes; D7, selection of the reported result. Surguladze et al. (2007) [34] and (2011) [35] report the same patient sample with different control groups.

Table S3. Information sources, search strings as run, date of execution and number of records retrieved.

Source	Search string as run	Date	Records
PubMed/MEDLINE	("long-acting injectable"[tiab] OR "long acting injectable"[tiab] OR "long-acting antipsychotic"[tiab] OR "long acting antipsychotic"[tiab] OR "long-acting antipsychotics"[tiab] OR "long acting antipsychotics"[tiab] OR "depot antipsychotic"[tiab] OR "depot antipsychotics"[tiab] OR LAI[tiab] OR "paliperidone palmitate"[tiab] OR "risperidone long- acting"[tiab] OR "Risperdal Consta"[tiab] OR "risperidone ISM"[tiab] OR "aripiprazole once-monthly"[tiab] OR "aripiprazole lauroxil"[tiab] OR "olanzapine pamoate"[tiab] OR "Delayed-Action Preparations"[Mesh]) AND (MRI[tiab] OR "magnetic resonance"[tiab] OR neuroimaging[tiab] OR "brain imaging"[tiab] OR "diffusion tensor"[tiab] OR DTI[tiab] OR "cortical thickness"[tiab] OR "grey matter"[tiab] OR "gray matter"[tiab] OR "white matter"[tiab] OR "intracortical myelin"[tiab] OR "voxel-based morphometry"[tiab] OR "brain volume"[tiab] OR "Magnetic Resonance Imaging"[Mesh] OR "Diffusion Tensor Imaging"[Mesh] OR "Neuroimaging"[Mesh]) AND (schizophreni*[tiab] OR "first-episode psychosis"[tiab] OR "first episode psychosis"[tiab] OR "Schizophrenia"[Mesh] OR "Psychotic Disorders"[Mesh]) AND english[Filter] AND humans[Filter]	13 Aug 2026	55
Scopus	TITLE-ABS-KEY ("long-acting injectable" OR "long acting injectable" OR "long-acting antipsychotic*" OR "long acting antipsychotic*" OR "depot antipsychotic*" OR "paliperidone palmitate" OR "risperidone long-acting" OR "Risperdal Consta" OR "risperidone ISM" OR "aripiprazole once-monthly" OR "aripiprazole lauroxil" OR "olanzapine pamoate") AND TITLE-ABS-KEY (MRI OR "magnetic resonance" OR neuroimaging OR "brain imaging" OR "diffusion tensor" OR DTI OR "cortical thickness" OR "grey matter" OR "gray matter" OR "white matter" OR "intracortical myelin" OR "voxel-based morphometry" OR "brain volume") AND TITLE-ABS-KEY (schizophreni* OR"first-episode psychosis") AND (LIMIT-TO (LANGUAGE , "English"))	13 Aug 2026	125
Cochrane CENTRAL	Advanced search, Title Abstract Keyword, limited to Trials: (schizophreni* OR schizoaffective OR psychosis OR psychotic OR "first episode psychosis") AND ("long acting injectable" OR "long-acting injectable" OR "long acting antipsychotic" OR "long-acting antipsychotic" OR "depot antipsychotic" OR "depot injection" OR "depot neuroleptic" OR LAI OR "once monthly" OR "three monthly" OR "six monthly" OR "paliperidone palmitate" OR "Invega Sustenna" OR "Invega Trinza" OR "Invega Hafyera" OR "risperidone microsphere" OR "Risperdal Consta" OR "risperidone ISM" OR Okedi OR Perseris OR Uzedy OR Rykindo OR "aripiprazole monohydrate" OR "aripiprazole lauroxil" OR "Abilify Maintena" OR Aristada OR "olanzapine pamoate" OR "Zyprexa Relprevv" OR "fluphenazine decanoate" OR "haloperidol decanoate" OR "flupenthixol decanoate") AND (MRI OR fMRI OR "magnetic resonance" OR neuroimaging OR "brain imaging" OR "brain volume" OR "diffusion tensor" OR DTI OR "fractional anisotropy" OR "mean diffusivity" OR tractograph* OR"cortical thickness" OR "cortical thinning" OR "grey matter" OR "gray matter" OR "white matter" OR myelin* OR morphometr* OR "voxel based" OR VBM OR "resting state" OR "functional connectivity" OR "regional homogeneity")	13 Aug 2026	80
Google Scholar, searched as a supplementary source via Publish or Perish	Three queries run separately, without truncation, patents and citations excluded, first 200 results by relevance screened for each query: (1) "long-acting injectable" schizophrenia MRI OR neuroimaging OR "cortical thickness"; (2) "paliperidone palmitate" OR "risperidone long-acting" schizophrenia "white matter" OR "gray matter" OR myelin; (3) "depot antipsychotic" schizophrenia "magnetic resonance" OR "diffusion tensor". The three queries returned 20, 68 and 30 records, respectively	13 Aug 2026	118
ClinicalTrials.gov and WHO ICTRP	Condition: schizophrenia; intervention: long-acting injectable OR paliperidone palmitate OR risperidone long-acting OR aripiprazole once- monthly OR olanzapine pamoate; outcome measures screened for any neuroimaging endpoint	13 Aug 2026	Two completed trials with prespecified imaging endpoints identified and

Source	Search string as run	Date	Records
			discussed in Sections 4.7 and 4.8
Citation tracking, Scopus	Backward and forward citation searching of all included studies: 175 citing records and 244 cited references	13 Aug 2026	419 identified, 48 already retrieved, 371 screened
Total	Databases and registers: 378 records, 62 duplicates removed, 316 screened. Other methods: 371 screened. 687 unique records screened, 24 reports assessed in full text, 9 studies included in 10 reports	13 Aug 2026	687

All searches were run on 13 August 2026 with no lower date limit. Embase, PsycINFO and Web of Science were not searched because of the lack of institutional access, which is reported among the limitations of the review.

Table S4. PRISMA 2020 checklist, based on the PRISMA 2020 statement [80]

	Checklist item	Location where the item is reported
1	Title: identify the report as a systematic review	Title page
2	Abstract: see PRISMA 2020 for Abstracts checklist	Abstract
3	Rationale for the review in the context of existing knowledge	Section 1
4	Explicit statement of the objectives or questions addressed	Section 1, final paragraph
5	Inclusion and exclusion criteria and how studies were grouped	Section 2.2
6	Information sources, with the date each was last searched	Section 2.3; Table S3
7	Full search strategy for all databases and registers	Table S3
8	Selection process, number of reviewers, use of automation tools	Section 2.4, including disclosure of the automated pre- classification used to assist screening
9	Data collection process	Section 2.5
10a	Outcomes for which data were sought	Sections 2.2 and 2.5; Table 2
10b	Other variables for which data were sought	Section 2.5; Table 1
11	Study risk of bias assessment	Section 2.6; Tables S1 and S2
12	Effect measures	Not applicable: narrative synthesis without pooled effect measures, Section 2.7
13a	Processes for deciding which studies were eligible for each synthesis	Sections 2.2 and 2.7
13b	Methods to prepare the data for presentation or synthesis	Section 2.7
13c	Methods used to tabulate or visually display results	Section 2.7; Tables 1 and 2
13d	Methods used to synthesize results	Section 2.7: narrative synthesis, no meta-analysis
13e	Methods used to explore causes of heterogeneity	Not applicable
13f	Sensitivity analyses	Not performed; the small number of studies is reported in Section 4.9
14	Reporting bias assessment	Section 4.9: funnel plot asymmetry could not be assessed; unpublished registered trials are discussed
15	Certainty assessment	Not performed; reported as a limitation in Section 4.9
16a	Results of the search and selection process	Section 3.1; Figure 1
16b	Studies excluded at full text, with reasons	Section 3.1; Figure 1
17	Characteristics of the included studies	Tables 1 and 2
18	Risk of bias in the included studies	Section 3.2; Figures 2 and 3; Tables S1 and S2
19	Results of individual studies	Table 2; Sections 3.3 to 3.7
20a-d	Results of syntheses	Sections 3.3 to 3.7; items on pooled estimates, heterogeneity and sensitivity analyses not applicable
21	Reporting biases	Section 4.9
22	Certainty of evidence	Not assessed formally; discussed qualitatively in Sections 4.9 and 5
23a	General interpretation of the results	Sections 4 and 5
23b	Limitations of the evidence included in the review	Section 4.9

	Checklist item	Location where the item is reported
23c	Limitations of the review processes used	Section 4.8, including the absence of protocol registration, the restriction to English-language reports, the databases that could not be searched and the absence of a formal certainty assessment
23d	Implications for practice, policy and future research	Sections 4.9 and 5
24a	Registration and protocol	Section 2.1: the review was not registered
24b	Where the protocol can be accessed	Not applicable
24c	Amendments to information provided at registration	Not applicable: the review was not registered
25	Support and sponsorship	Funding statement
26	Competing interests	Conflicts of Interest statement
27	Availability of data, code and other materials	Data Availability Statement; screening and extraction files available from the corresponding author on request
